# Sleep quality differentially modulates neural oscillations and proteinopathy in Alzheimer's disease

**DOI:** 10.1016/j.ebiom.2023.104610

**Published:** 2023-05-12

**Authors:** Maggie P. Rempe, Alex I. Wiesman, Daniel L. Murman, Pamela E. May, Nicholas J. Christopher–Hayes, Sara L. Wolfson, Craig M. Johnson, Tony W. Wilson

**Affiliations:** a Institute for Human Neuroscience, Boys Town National Research Hospital, Boys Town, NE, 68010, USA; b University of Nebraska Medical Center (UNMC), College of Medicine, Omaha, NE, 68198, USA; c Montreal Neurological Institute, McGill University, Montreal, Quebec, H3A 0G4, Canada; d Center for Mind and Brain, University of California, Davis, CA, 95618, USA; e Department of Pharmacology and Neuroscience, Creighton University, Omaha, NE, 68178 USA

**Keywords:** Magnetoencephalography, Resting-state, Delta, MEG, Mild cognitive impairment

## Abstract

**Background:**

Alterations in resting-state neural activity have been reported in people with sleep disruptions and in patients with Alzheimer's disease, but the direct impact of sleep quality on Alzheimer's disease-related neurophysiological aberrations is unclear.

**Methods:**

We collected cross-sectional resting-state magnetoencephalography and extensive neuropsychological and clinical data from 38 biomarker-confirmed patients on the Alzheimer's disease spectrum and 20 cognitively normal older control participants. Sleep efficiency was quantified using the Pittsburgh Sleep Quality Index.

**Findings:**

Neural activity in the delta frequency range was differentially affected by poor sleep in patients on the Alzheimer's disease spectrum. Such neural changes were related to processing speed abilities and regional amyloid accumulation, and these associations were mediated and moderated, respectively, by sleep quality.

**Interpretation:**

Together, our results point to a mechanistic role for sleep disturbances in the widely reported neurophysiological aberrations seen in patients on the Alzheimer's disease spectrum, with implications for basic research and clinical intervention.

**Funding:**

10.13039/100000002National Institutes of Health, USA.

**Research in context**

**Evidence before this study**

The literature was reviewed using a search of online databases (e.g., PubMed). Several studies describe long-term sleep disruption, protein deposition, and aberrant neuronal dynamics in Alzheimer's disease, but little is known about the how these effects intersect.

**Added value of this study**

This study shows differential effects of sleep disturbances on resting-state neural activity in patients on the Alzheimer's disease spectrum relative to healthy adults. Further, these disease-specific neural relationships impact cognitive abilities and interact with regional amyloid accumulation. Our findings suggest a key role of sleep disturbances in the neurophysiological changes seen in patients on the Alzheimer's disease spectrum.

**Implications of all the available evidence**

Understanding the complex interactions between sleep quality, Alzheimer's related pathologies, and cognitive functions could lead to noninvasive markers for early detection, more sensitive outcome metrics to gauge therapeutic interventions, and even modifiable targets for neuromodulatory and pharmacological therapies in Alzheimer's disease.

## Introduction

Changes in sleep patterns are common in healthy aging, however, extended periods of poor sleep have also been associated with increased risk for a myriad of health conditions, including Alzheimer's disease.^1^ Sleep disturbances are reported in up to 45% of patients with Alzheimer's disease,^2^^,^^3^ and individuals with sleep problems in middle-to-late adulthood have nearly a two-fold greater risk of developing cognitive impairments.^1^ It is likely that these sleep alterations interact with, and potentially accentuate, Alzheimer's disease symptomatology and neuropathology. As such, it is critical to understand how sleep disturbances contribute to the neurophysiological and neuropathological alterations seen in patients on the Alzheimer's disease spectrum. Understanding these complex interactions could lead to noninvasive markers for early detection and/or sensitive outcome metrics to gauge the efficacy of therapeutic interventions in early-stage Alzheimer's disease.

It is hypothesized that sleep has a bidirectional relationship with the development of Alzheimer's disease. On the one hand, sleep disturbances are thought to contribute to the development of Alzheimer's disease. Studies in mouse models have shown chronic sleep deprivation leads to significantly increased amyloid β (Aβ) deposition.^4^ Further, shorter sleep duration in humans has been associated with increased Aβ burden and cognitive deficits in older adults.^5^ On the other hand, Alzheimer's disease pathology is thought to exacerbate sleep disruption: both regional atrophy and Aβ accumulation are related to alterations in sleep architecture.^6^, ^7^, ^8^ Considering this bidirectional loop between sleep disturbances and Alzheimer's disease pathology, as well as the well-described relationship between sleep alterations and cognitive performance, it follows that sleep disturbances may also relate to Alzheimer's disease symptomatology.

There is an abundance of neuroimaging studies showing alterations in resting-state neural activity in patients with Alzheimer's disease. These studies have broadly shown changes in functional connectivity, particularly in dorsal attention and default mode networks.^9^, ^10^, ^11^ Similar alterations in spontaneous neural activity have been reported in those with sleep disruptions.^12^, ^13^, ^14^, ^15^ Analyses of spontaneous neural activity have also demonstrated spectrally-specific disturbances,^16^, ^17^, ^18^, ^19^, ^20^, ^21^, ^22^ particularly in the alpha (8–12 Hz) band, which relate to proteinopathy and impaired cognitive abilities in patients with Alzheimer's disease.^16^^,^^20^, ^21^, ^22^

Given these complementary findings, it is surprising that the intersection of long-term sleep disruption, proteinopathy, and their net impact on the neuronal dynamics underlying cognitive function in Alzheimer's disease remains an underexplored frontier. Using an extensive neuropsychological and clinical battery alongside quantitative whole-brain Aβ PET and advanced magnetoencephalographic (MEG) imaging, we quantify the impact of sleep disturbances and Alzheimer's disease-related proteinopathy on spectrally specific neural activity in 38 biomarker-confirmed patients on the Alzheimer's disease spectrum and 20 demographically-matched cognitively-normal older adults. We hypothesized that disease-related abnormalities in spontaneous neural activity would relate to sleep quality, and that these effects would mediate the relationship between neural activity and cognitive impairments. Further, we expected that the impact of regional Aβ deposition on neural activity would be moderated by sleep quality, indicating stronger neural effects of Alzheimer's disease proteinopathy in patients with worse sleep disturbances.

## Methods

### Participants

A total of 64 participants were enrolled in this study, 44 of whom were diagnosed with amnestic mild cognitive impairment (*n* = 21) or mild probable Alzheimer's disease (*n* = 23), as determined by a fellowship-trained neurologist specializing in memory disorders. The remaining 20 participants were cognitively normal older adults who reported no amnestic or cognitive complaints. We did not perform an explicit power analysis to determine sample size, as this was a novel comparison of sleep quality and whole-brain resting state metrics in the setting of AD, with little-to-no comparable previous literature from which to extract relevant effect sizes. However, we designed our study to collect data from a sample size that is comparable to (and often far above) what is typically seen in the field of MEG Alzheimer's disease studies.^19^ Exclusionary criteria included any medical illness affecting the central nervous system, any neurological or psychiatric disorder (other than mild cognitive impairment or Alzheimer's disease), history of head trauma, current substance use disorder, and standard MEG exclusion criteria (e.g., ferromagnetic implants).

### Ethics

The Institutional Review Board at the University of Nebraska Medical Center approved the study (protocol #302-18-FB), and all research protocols complied with the Declaration of Helsinki. Written informed consent was obtained from each participant after a full description of the study. Patient informants were also required to be present for each participant on the Alzheimer's disease spectrum (i.e., those with amnestic mild cognitive impairment or mild probable Alzheimer's disease) to ensure their comfort over the course of the study, and as such, informed consent was obtained from each informant as well. For patients whose capacity to consent was questionable, informed assent was obtained from the research participant in addition to informed consent from a legally authorized representative.

### Neuropsychological testing

All participants underwent extensive neuropsychological testing in collaboration with a clinical neuropsychologist specializing in memory disorders, which has been described in detail elsewhere.^20^^,^^21^ Raw scores for each participant were converted to demographically adjusted z-scores based on published normative data.^23^, ^24^, ^25^, ^26^ These demographically corrected z-scores per test were then averaged to create composite cognitive domain z-scores per participant. Due to the strong relationship between delta-frequency neural activity and processing speed abilities reported previously in these patients, in addition to previously reported relationships between sleep quality and processing speed in Alzheimer's disease and community-dwelling older adults,^20^^,^^27^^,^^28^ we focused on the processing speed composite scores for this study. In addition, participants completed the Pittsburgh Sleep Quality Index (with the help of an informant, if necessary) to assess sleep quality.^29^ From these data, sleep efficiency was calculated by dividing the reported time asleep by reported time in bed.^30^

### MEG data acquisition and preprocessing

MEG recordings took place in a one-layer magnetically shielded room (MSR) with active shielding engaged for environmental noise compensation. Participants were seated in a nonmagnetic chair within the MSR, with their head positioned within the sensor array. A 306-sensor MEGIN MEG system (Helsinki, Finland), equipped with 204 planar gradiometers and 102 magnetometers, was used to sample neuromagnetic responses continuously at 1 kHz with an acquisition bandwidth of 0.1–330 Hz. Participants were instructed to rest with their eyes closed for 8 min^31^ and were monitored by a real-time audio-video feed from inside the shielded room throughout MEG data acquisition. MEG data acquisition and processing is further described in the Supplementary Methods (S1.1–S1.4)*.* All preprocessing, source imaging, and power spectral density (PSD) computations were performed in *Brainstorm*.^32^

### MEG source imaging and frequency power maps

Source analysis of neuromagnetic fields used an overlapping-spheres forward model, unconstrained to the cortical surface. A linearly constrained minimum variance (LCMV) beamformer implemented in *Brainstorm* was used to spatially filter the epoched data based on the data covariance computed from the resting-state recording. These source-level time series data were then transformed into the frequency-domain using Welch's method for estimating PSD (window = 1 s; 50% overlap), averaged over canonical frequency bands (delta: 2–4 Hz; theta: 5–7 Hz; alpha: 8–12 Hz; beta: 15–29 Hz), and these spectral maps were normalized to the total power of the frequency spectrum. The norm of the three unconstrained orientations per location (i.e., vertex) and map were then projected onto a standardized template surface (including the cerebellum) for statistical modelling. Finally, frequency-wise maps of Alzheimer's disease-related deviations in spontaneous neural activity were created by z-scoring the whole-brain power maps of patients on the Alzheimer's disease spectrum to maps of the spectral power means and standard deviations from the control sample. The Desikan-Killiany (DK) atlas^33^ was used to extract the average z-scored relative power values for each patient per each of the 68 atlas regions, and these values were used for additional statistical modelling.

### Florbetapir 18F positron emission tomography

Combined PET/computed tomography (CT) data using^18^F-florbetapir (Amyvid, Eli Lilly) and a GE Discovery MI digital scanner were collected following the standard procedures described by the Society of Nuclear Medicine and Molecular Imaging (3D acquisition; single intravenous slow-bolus <10 mL; dose = 370 MBq; waiting period = 30–50 min; acquisition = 10 min).^34^ Images were attenuation corrected using the CT data, reconstructed in MIMneuro (slice thickness = 2 mm),^35^ converted to voxel standardized uptake values (SUV) based on body weight, and normalized into Montreal Neurological Institute space. Each scan was read by a fellowship-trained neuroradiologist blinded to group assignment and assessed as being “amyloid-positive” or “amyloid-negative” using established clinical criteria.^35^ At this stage, patients who were amyloid-negative were excluded from the Alzheimer's disease spectrum group (no controls were amyloid positive). Images were then normalized to the crus of the cerebellum (SUIT template)^36^ to generate voxel-wise maps of SUV ratios (SUVR),^37^ and back-transformed into each patient's native MRI space using their FreeSurfer-processed T1 data. The PET data overlapping with each individual's cortical gray-matter ribbon was then projected onto a tessellated average template surface using mri_vol2surf (maximum value; projection fraction = 1; steps of 2).^38^ Finally, the DK atlas was used to extract average SUVR values for each participant per each of the 68 atlas regions to be used for statistical modelling.

### Statistical analysis and visualization

Whole-brain statistical analyses were performed on the source-imaged MEG data using a general linear approach in SPM12, with multiple comparisons controlled using threshold-free cluster enhancement (TFCE; *p*_FWE_ < 0.05; see Supplementary Methods S1.4).^39^ Initial tests investigated the interaction between group (controls vs. Alzheimer's disease spectrum) and sleep efficiency on resting-state power in each frequency band. Data were extracted from the vertex exhibiting the strongest relationship in each significant cluster and plotted using ggplot2^40^ for interpretation of directional effects and further statistical analyses. Tobit regression modelling was used to ensure the initial results were unbiased by right-censoring of the sleep efficiency data (see Supplementary Methods S1.5). To test for lateralization of these effects, we computed a laterality index ([left – right]/[left + right]) of spontaneous power at each relevant cluster peak (using the mirrored location across the longitudinal fissure) and tested for interactive effects of sleep efficiency and group on these indices using linear regression.

Linear regression models were computed in *R*^41^ to test for evidence of a potential mediation (i.e., indirect) effect of sleep efficiency on the relationship between resting-state activity and processing speed abilities in patients on the Alzheimer's disease spectrum. This was performed using the extracted peak of the strongest relationship identified from the TFCE statistical maps. Other subpeaks identified from the TFCE output in SPM12 were also extracted to determine spatial specificity of the tested mediation. A causal directed acyclic graph (cDAG) was used to identify potential confounders (see Fig. S1 in the Supplemental Methods). Mediation models were then performed to assess this indirect effect with 10,000 iterations of bias-corrected bootstrapping to test for significance using the *mediation* package in *R*.^41^, ^42^, ^43^ Finally, to explore the interactive effect of sleep efficiency on the relationship between regional Aβ uptake and disease-related changes in spontaneous neural activity, linear mixed-effects modelling was performed using the *nlme* package^44^^,^^45^ in R with the following form: *Relative Spectral Power ∼ SUVr∗efficiency + Age, random = (∼1 | Patient),* assuming a normal Gaussian distribution of random effects*.* Of note, the interaction term (i.e., SUVr∗efficiency) also includes main effect terms (i.e., main effects of SUVr and efficiency) by default. Assumptions of linear mixed-effects models were tested and found to have no violations (normality, homoscedasticity, and linearity)*.* This model was tested in each frequency band that showed group-by-sleep efficiency interactions in the whole-brain analyses described above. Age was included as a nuisance covariate in all statistical analyses.

### Role of the funding source

The funders had no role in study design, data collection, analysis, decision to publish, or manuscript preparation.

## Results

Participant exclusions are detailed in the Supplementary Results (S2.1). The results presented here include the final sample of 38 biomarker-confirmed patients on the Alzheimer's disease spectrum (M_age_ = 69.21, SD = 6.91) and 20 older adults with normal cognition (19 amyloid-negative and one without PET; M_age_ = 72.70, SD = 4.73). Group demographics can be found in Table 1 for cognitively normal controls and Alzheimer's disease spectrum groups, and in Table S3 for the amnestic mild cognitive impairment and Azheimer's disease subgroups. The cognitively normal controls and Alzheimer's disease spectrum groups were matched on key demographics except age, which was included as a nuisance covariate in all statistical modelling.

**Table 1:** Descriptive statistics by group.

Descriptives, mean (SD)	Cognitively normal controls	Alzheimer's disease spectrum	*p - value*^a^
	n = 20	n = 38	
Age, years	72.70 (4.73)	69.21 (6.91)	0.028
Education, years	16.60 (2.87)	15.50 (2.72)	0.166
Sex, female (%)	12 (60%)	18 (47%)	0.360
Handedness, Left (%)	1 (5%)	3 (8%)	0.679
Processing speed (Z-score)	0.66 (0.83)	−0.90 (1.42)	<0.001
Sleep efficiency, %	89.47 (12.73)	95.79 (7.54)	0.051
MMSE	29.40 (0.88)	24.16 (3.77)	<0.001

a *p*-values indicate group differences as measured by independent samples *t*-test with equal variances not assumed (age, education, processing speed, sleep efficiency, and MMSE) or chi-square test (sex and handedness).

### Sleep efficiency and disease status have interactive effects on resting-state neural activity

**Fig. 1:**
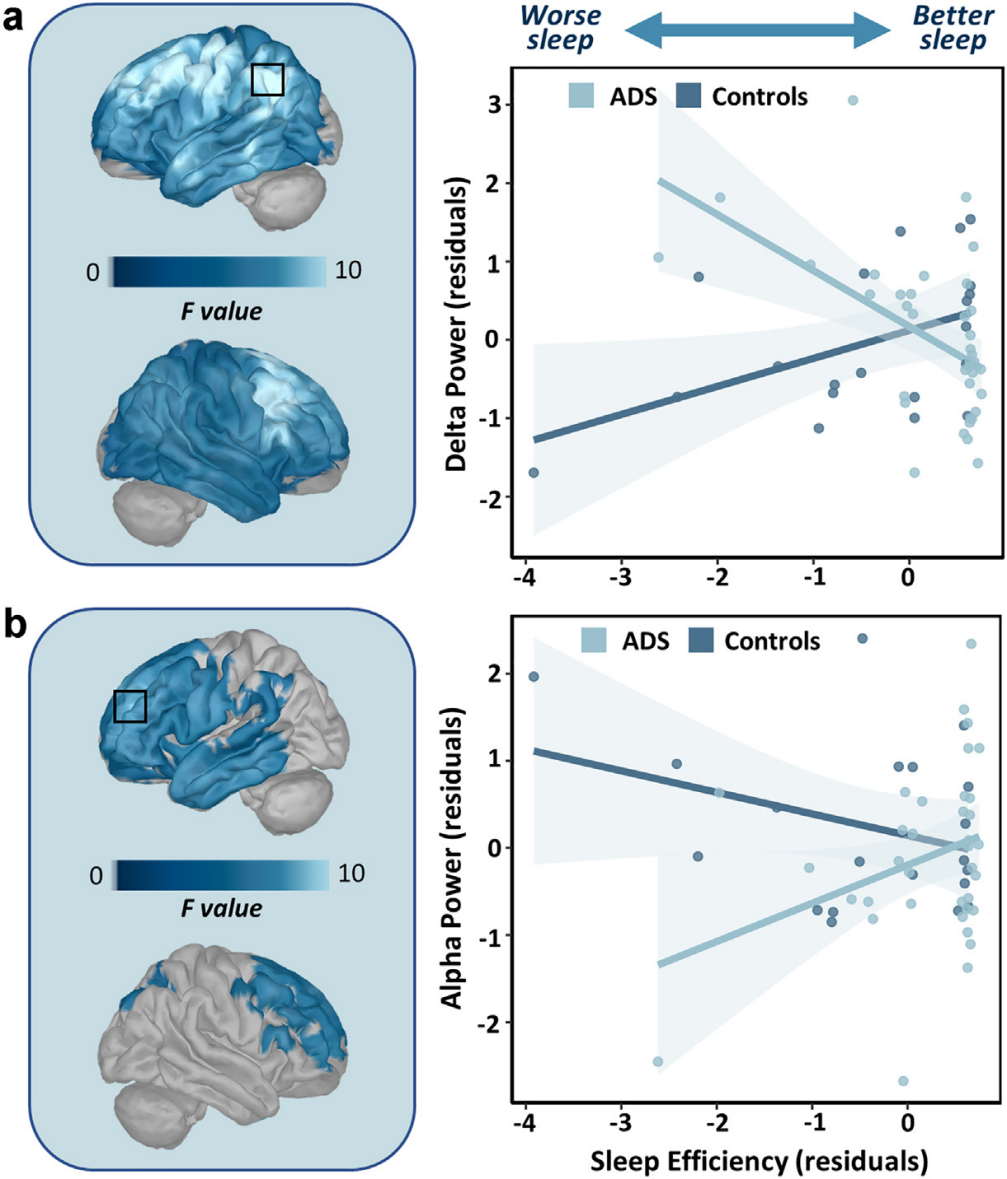
**Interactive effects of disease status and sleep efficiency on spontaneous power**. General linear analyses revealed significant group-by-sleep efficiency interactions, controlling for age, in the delta (a; 2–4 Hz, n = 57) and alpha (b; 8–12 Hz, n = 58) frequency bands. Surface maps to the left indicate significant clusters, which were corrected for multiple comparisons using a stringent threshold-free cluster enhancement approach (*p*_*FWE*_ < 0.05), with the box indicating the peak of the relationship. Delta **(a)** and alpha **(b)** power values were extracted from the peak of the cluster (indicated by the black box) and used to visualize the direction and nature of these interactive effects in the scatterplots to the right of each map. Shaded areas indicate 95% Confidence Intervals.

Significant interactions between sleep efficiency and disease status (i.e., Alzheimer's disease spectrum vs controls) were identified in the delta and alpha frequency bands (Fig. 1). The interactive effect on delta power extended across frontotemporal cortices, with the strongest effect in the left supramarginal gyrus (*F_peak_* = 13.44, *p_FWE_* = 0.011). This effect was such that, in the Alzheimer's disease spectrum group, delta power increased with worse sleep efficiency, while the opposite effect was present in the control sample. Interactive effects on alpha power were identified primarily in frontal and anterior temporal regions, with the peak effect in the left superior frontal gyrus (*F_peak_* = 8.91, *p_FWE_* = 0.026). In contrast to the delta band, this interaction was such that, in the Alzheimer's disease spectrum group, alpha power decreased with worse sleep efficiency, with the opposite effect in the controls. Both the delta *(p* = .001) and alpha (*p* = .002) interaction effects remained when using tobit regression models to account for the right-censoring of the sleep efficiency data. Additionally, when controlling for the local aperiodic exponent and offset at the peak, both the delta (*p* < .001) and alpha (*p* = .005) interaction effects remained significant. None of these effects were significantly lateralized to one hemisphere (alpha: *p* = .819; delta: *p* = .927).

### The relationship between delta activity and processing speed in Alzheimer's disease is fully mediated by sleep disturbances

**Fig. 2:**
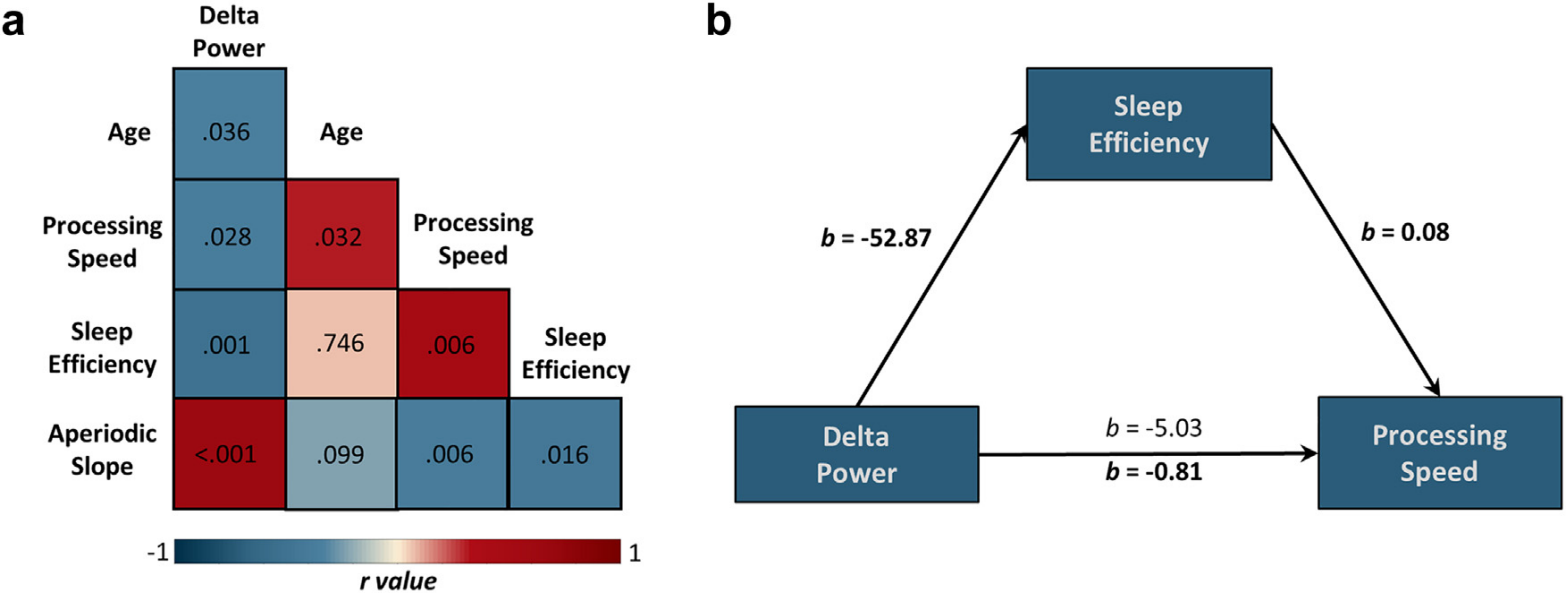
**Sleep efficiency fully mediates the relationship between delta power and processing speed in patients on the Alzheimer's disease spectrum. a)** Correlation matrix showing relationships between all variables of interest. Red indicates a positive relationship while blue indicates a negative relationship. Values in each box indicate the *p*-value of the Pearson correlation (n = 37). **b)** Mediation model path showing the sleep efficiency mediation of associations between delta power and processing speed, controlling for age. All beta estimates are unstandardized. The pre-mediator model is indicated by the beta above the arrow while the beta below the arrow indicates the post-mediator model. The model indicated full mediation, with a significant indirect effect.

Given the strong negative relationship identified between delta power and sleep efficiency in the Alzheimer's disease spectrum group, as well as previously-reported relationships between delta power, sleep quality, and processing speed in Alzheimer's disease and community-dwelling older adults,^20^^,^^27^^,^^28^ we examined whether this delta-sleep effect accounted for processing speed deficits in our study. Specifically, we tested whether the relationship between delta power and processing speed was mediated by sleep efficiency in patients on the Alzheimer's disease spectrum. A full matrix of correlations between the variables of interest can be found in Fig. 2 and Table S1. Sleep efficiency fully mediated the relationship between delta power and processing speed, such that there was a significant indirect effect (Average Causal Mediated Effect; ACME = −4.23, 95% CI [−9.71, −0.91], *p* = .010; Fig. 2), and no significant direct relationship between delta power and processing speed (Average Direct Effect; ADE = −0.81, 95% CI [−5.29, 7.10], *p* = .820; Fig. 2) once sleep efficiency was included in the model. Fig. 2 illustrates the associations between delta power, processing speed, and sleep efficiency. A table detailing path estimates for the full mediation model can be found in the Supplementary Results (Table S2). The pattern of results in these mediation analyses were virtually unchanged when controlling for the aperiodic exponent and offset (see Supplementary Results S2.2). To test for spatial specificity of this mediation effect, subpeaks were identified in the TFCE output from SPM12 (Fig. 1) and delta power values were extracted from the right and left frontal cortices. These frontal peaks did not exhibit a significant relationship with processing speed, but did show a significant indirect effect on processing speed via sleep efficiency (Right: ACME = −3.58, 95% CI [−7.81, −0.77], *p* = .012; Left: ACME = −5.11, 95% CI [−11.82, −1.06], *p* = .013), indicating that this mediation effect may not be specific to the left supramarginal gyrus.

### Sleep quality moderates the relationship between regional Aβ uptake and Alzheimer's disease-related delta power deviations

**Fig. 3:**
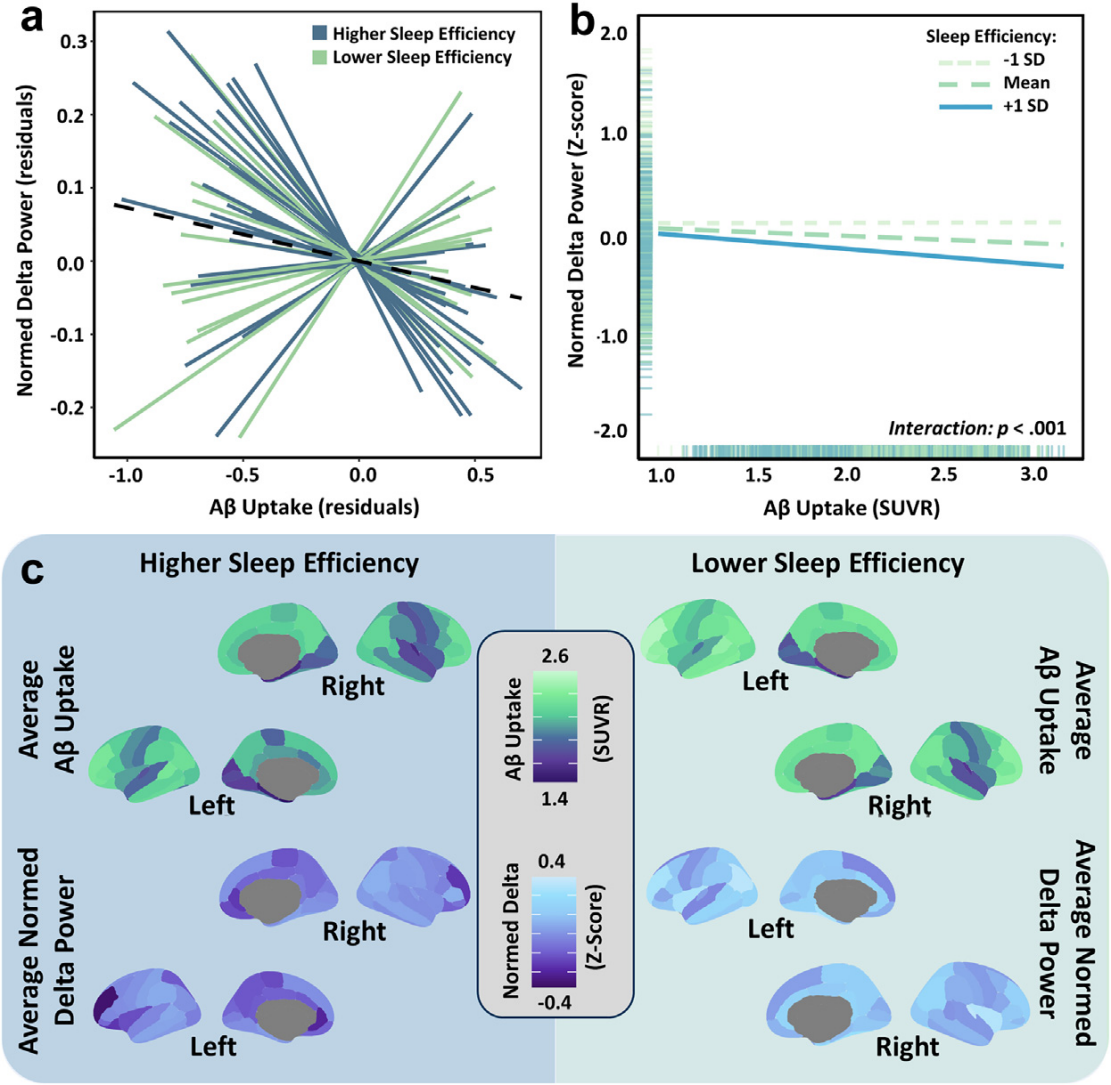
**Sleep efficiency moderates the relationship between regional Aβ uptake and Alzheimer's disease-related delta-frequency neural deviations. a)** Linear mixed models (n = 37) assessed the spatial correspondence between regional Aβ accumulation and deviations from healthy levels of spontaneous delta power in patients on the Alzheimer's disease spectrum. Delta power estimates for patients on the Alzheimer's disease spectrum were normalized to the mean and standard deviation of comparable estimates from the control participants, resulting in z-score deviations from healthy levels. Each line represents the individual nested model fit between Aβ uptake and delta power for each patient (i.e., the relationship between Aβ uptake and delta power across the full cortex for each participant), with the overall model fit overlaid in black. Sleep efficiency scores were included in the model as a continuous variable and subgroups (defined using a median split) were only for visualization purposes. The color of each line indicates higher (i.e., blue; median split) or lower (i.e., green) sleep efficiency scores. **b)** Plot displaying the significant interaction between sleep efficiency and Aβ accumulation on delta power. Each line represents the relationship between Aβ accumulation and delta power at varying levels of sleep efficiency (i.e., −1 SD, mean, and +1 SD). Rug plots on each axis display the distribution of the variables in native units. **c)** Average regional Aβ uptake, measured by quantitative ^18^F florbetapir PET in SUVRs and average normed regional delta power (i.e., deviations from control levels of spontaneous delta power) in patients on the Alzheimer's disease spectrum with higher or lower sleep efficiency (defined using a median split).

Sleep efficiency significantly moderated the relationship between regional Aβ uptake and deviations from healthy levels of spontaneous delta power (*b* = −0.010, 95% CI [−0.015, −0.005], *t*(2477) = −3.94; *p* < .001), such that those with better sleep efficiency exhibited a strong negative relationship between Aβ uptake and disease-related spontaneous delta power, while those with poor sleep efficiency had no such relationship (Fig. 3). These effects remained significant when controlling for the regional aperiodic exponent and offset (*b* = −0.009, 95% CI [−0.014, −0.004], *t*(2475) = −3.88; *p* < .001). In other words, contrary to our predictions, cortical regions with higher levels of Aβ accumulation exhibited reduced spontaneous delta power relative to controls, but *only in patients with better sleep quality*.

## Discussion

Aberrations in resting-state neural activity in patients on the Alzheimer's disease spectrum have been extensively explored in the literature,^9^, ^10^, ^11^ and more recent studies have begun to show similar resting-state alterations related to sleep disturbances.^12^, ^13^, ^14^, ^15^ However, the interaction of Alzheimer's disease pathology and sleep disturbances on neural oscillatory activity has not been studied. In this study, we show that relationships between resting-state neural activity and sleep quality are altered by Alzheimer's disease and Alzheimer's disease-related pathology. Additionally, we found that the relationship between delta-frequency neural activity and processing speed abilities in patients on the Alzheimer's disease spectrum is fully mediated by sleep efficiency. Finally, and contrary to our original hypotheses, we show that patients with better sleep quality exhibit a stronger relationship between delta-frequency neural deviations and regional amyloid burden, indicating a potential compensatory mechanism. These data suggest a key influence of sleep disturbances on previously-reported relationships between Alzheimer's disease-related proteinopathy, neurophysiological changes, and cognitive impairments, which should be more thoroughly considered in future research.

There is strong agreement in the literature that Alzheimer's disease patients exhibit a neural slowing effect: decreases in high-frequency power (i.e., alpha and beta) and concurrent increases in low-frequency oscillations (i.e., delta and theta)^9^^,^^10^^,^^20^^,^^21^^,^^46^ that relate to Alzheimer's disease proteinopathy and cognitive impairments.^16^^,^^21^^,^^22^^,^^47^ Our results showed that patients on the Alzheimer's disease spectrum who had better sleep efficiency tended to have higher levels of alpha power in frontotemporal regions and lower levels of delta power in temporoparietal areas, while the cognitively normal controls showed opposite effects. In the delta band in particular, we further showed that the relationship between neural activity at the temporoparietal junction (i.e., the peak of the interactive effects of sleep and disease status) and processing speed abilities was fully mediated by sleep efficiency in patients on the Alzheimer's disease spectrum. This indicates that the commonly-reported decreases in frontal alpha power and increases in posterior delta power in patients on the Alzheimer's disease spectrum, along with their deleterious effects on cognition, may be, at least to some extent, related to sleep disturbances. However, further research is needed to determine whether this represents a neuroprotective effect of sleep against pathological neural slowing, or rather a detrimental effect of Alzheimer's disease-related neurophysiological changes on sleep quality.

Unexpectedly, we also found that disease-associated increases in delta activity were lower in regions with higher Aβ burden, which initially appeared to contradict previous findings.^21^^,^^22^ However, this relationship was linearly moderated by sleep quality across patients, such that those who reported better sleep efficiency exhibited the strongest negative effect. This finding tentatively suggests that higher sleep efficiency may have a neuroprotective effect, such that better sleep is key to maintaining more normal levels of delta oscillatory activity in brain regions with higher Aβ burden. These relationships should be further studied in the future, especially in the setting of more objective sleep measurements.

While we cannot draw clear conclusions regarding the neurotransmitter systems involved in our findings, there is notable overlap in the disruption of cholinergic signaling in both Alzheimer's disease and sleep disorders. It is well-known that there is a loss of cholinergic activity in Alzheimer's disease, which is thought to underlie much of the common cognitive symptoms.^2^^,^^48^ This loss of cholinergic activity may also contribute to sleep disruptions, as cholinergic signaling is crucial for regulation of the sleep/wake cycle.^49^, ^50^, ^51^ The overlapping physiology of these cholinergic disruptions may be key to the altered neural dynamics observed in Alzheimer's disease, and future research might focus on the interplay between neurotransmitter systems, neural activity, sleep, and Alzheimer's disease proteinopathy.

Before closing, it is important to discuss the limitations of this study. First, while the Pittsburgh Sleep Quality Index is validated in the literature and commonly used to assess sleep quality, more objective methods (i.e., actigraphy or polysomnography) would provide a more complete understanding of these complex interactions than self-report metrics. Additionally, while resting-state neuroimaging paradigms have historically been beneficial in studying neural activity in patient populations, there are limitations to the conclusions that can be drawn using such data. Analyses targeting the impacts of sleep quality on oscillatory activity during active cognitive processing (i.e., task-based paradigms) would provide valuable insight and would shed light on the complex interplay between disease mechanisms, sleep disruptions, and cognitive impairments. It is also important to note the relatively limited sample size of this study. While typical MEG studies of patients on the Alzheimer's disease spectrum rarely exceed the sample size used here, the complex relationships explored in these analyses may be further elucidated in a larger sample. Additionally, an ideal study design would follow such patients longitudinally, to better characterize progressive cognitive decline and eventual conversion to Alzheimer's disease for patients with amnestic mild cognitive impairments. Though this design would suffer from its own limitations, including clinical progression of patients to the point that they can no longer participate in research, and inability to discern cause and effect of the relationships explored here given the bidirectional effects of sleep quality and development of Alzheimer's-related pathology.

These data suggest a differential effect of sleep disturbances on resting-state neural activity in patients on the Alzheimer's disease spectrum compared to cognitively-normal controls. Such neural changes, and particularly those observed in the slower delta band, exhibit relationships to cognitive abilities and regional amyloid accumulation that are mediated and moderated, respectively, by sleep quality. Taken together, our results point to a key role of sleep disturbances in the neurophysiological changes seen in patients on the Alzheimer's disease spectrum, and warrant consideration in future studies. An enhanced understanding of these complex interactions could lead to noninvasive markers for early detection, more sensitive outcome metrics to gauge therapeutic interventions, and even modifiable targets for neuromodulatory and pharmacological therapies in Alzheimer's disease.

## Contributors

Maggie P. Rempe: Conceptualization, Methodology, Formal Analysis, Investigation, Resources, Writing—Original Draft, Visualization. Alex I Wiesman: Conceptualization, Methodology, Software, Formal Analysis, Investigation, Resources, Supervision, Project Administration, Funding Acquisition, Writing—Review & Editing. Daniel L Murman: Conceptualization, Methodology, Resources, Writing—Review & Editing. Pamela E May: Conceptualization, Methodology, Resources, Writing—Review & Editing. Nicholas J. Christopher–Hayes: Software, Methodology, Writing—Review & Editing. Sara L Wolfson: Resources, Writing—Review & Editing. Craig M Johnson: Methodology, Investigation, Resources, Writing—Review & Editing. Tony W Wilson: Conceptualization, Methodology, Resources, Writing—Review & Editing, Supervision, Funding Acquisition. All authors have read and approved the final version of this manuscript. Authors MPR, AIW, and TWW have verified the underlying data.

## Data sharing statement

The data used in this article can be made available upon reasonable request.

## Declaration of interests

All authors declare no conflicts of interest. Dr. Murman reported receiving grants from Green Valley Pharmaceuticals, Functional Neuromodulation, Roche, and Eli Lilly and Co. and serving on an advisory board for Biogen.
