# Supplementary material for: Sleep quality differentially modulates neural oscillations and proteinopathy in Alzheimer's disease
Source: eBioMedicine. 2023 May 12;92:104610. doi: 10.1016/j.ebiom.2023.104610 (PMC10200835; doi:10.1016/j.ebiom.2023.104610)
Supplement: Supplementary material [file mmc1.pdf]

Supplementary Methods:

Sleep quality differentially modulates neural oscillations and proteinopathy in Alzheimer's disease (Rempe et al.)

S1.1 Structural MRI Acquisition, Processing, and MEG-MRI Coregistration

Prior to MEG acquisition, four coils were attached to the participants' heads and localized, together with the three fiducial points and scalp surface, using a 3-D digitizer (Fastrak 3SF0002, Polhemus Navigator Sciences, Colchester, VT, USA). Once the participant was positioned for MEG recording, an electrical current with a unique frequency label (e.g., 322 Hz) was fed to each of the coils. This induced a measurable magnetic field and allowed each coil to be localized in reference to the sensors throughout the recording session. Since coil locations were also known in head coordinates, all MEG measurements could be transformed into a common coordinate system. With this coordinate system (including the scalp surface points), each participant's MEG data were co-registered with their own high-resolution structural T1-weighted MRI data (Siemens Prisma 3T; 64-channel head coil; TR: 2.3 seconds; TE: 2.98 ms; flip angle: 9°; FOV: 256 mm; slice thickness: 1 mm; voxel size: 1 mm^3) using an iterative closest-point rigid-body registration in Brainstorm (September 3, 2020 distribution) [1] and, after visual inspection, these fits were manually corrected. Triangulated cortical surfaces were computed from the T1 MRI data using FreeSurfer recon_all [2] and imported into Brainstorm. Individual cortical surfaces (including the cerebellum) were down-sampled to ≈17,000 vertices for computation of the forward model for use in MEG source imaging.

S1.2 MEG Data Pre-Processing

Only the gradiometer data (204 channels) were used for this analysis. Each MEG dataset was individually corrected for head motion and subjected to noise reduction using the signal space separation method with a temporal extension (MaxFilter v2.2; correlation limit: 0.950; correlation window duration: 6 seconds). [3] Noise-reduced MEG data underwent standard data preprocessing procedures using the Brainstorm software. [1] MEG data were bandpass filtered between 1 and 200 Hz and notch filtered at 60, 120, and 180 Hz, and ocular and cardiac artifacts were identified using an automated identification algorithm, supplemented by visual inspection of their temporal and spatial topographies. From these topographies, signal-space projectors (SSPs) were generated and reviewed for each type of artifact, and those accounting for ocular and cardiac components were removed from the gradiometer data. Artifact-reduced MEG data were then epoched into non-overlapping blocks of 4 seconds and down-sampled to 500 Hz. Epochs still containing major artifacts (e.g., SQUID jumps) were excluded within each participant using the ∪ of standardized thresholds of ± 2.5 median absolute deviations from the median for signal amplitude and gradient. After exclusions, a mean of 98.90 (SD: 8.56) and 96.89 (SD: 7.93) epochs were included for further analysis for the control and Alzheimer's Disease spectrum groups, respectively. Importantly, there was no significant difference in the amount of data used between the two groups (p = .377).

S1.3 Estimation of the Power Spectral Aperiodic Exponent

The aperiodic exponents of the power spectra from peak vertices of interest were estimated using *specparam* [4] (*Brainstorm* Matlab version; frequency range = 2–30 Hz; Gaussian peak model; peak width limits = 0.5 –12 Hz;

maximum n peaks = 4; minimum peak height = 3 dB; proximity threshold = 2 standard deviations of the largest peak; fixed aperiodic; no guess weight). These estimates of the *1/f* spectral power slope were then included as nuisance covariates in post-hoc statistical models, to determine whether any significant effects were accounted for by shifts in the broadband frequency spectrum across participants.

S1.4 Correction for Spatial Multiple Comparisons

To account for non-uniform spatial autocorrelation in the data, avoid assumptions of parametric modeling, and avoid selecting arbitrary cluster-forming thresholds, threshold-free cluster enhancement (TFCE; E = 1.0, H = 2.0; 5000 permutations)[5] was performed, with multiple comparisons correction set to cluster-wise $p_{FWE} < .05$. Clusters surviving at this threshold were used to create logical masks that were applied to the original statistical contrasts (i.e., vertex-wise *F*-values) for visualization in Brainstorm.

S1.5 Tobit Modeling

To ensure that none of the whole-brain results computed in SPM12 were biased by the right-censored nature of the sleep efficiency data, we performed tobit regression modeling on the peak-vertex data from each significant relationship using the *AER* package[6] in *R*. These models were right-censored at 100 with a gaussian distribution, took the form: *Sleep Efficiency ~ Group * MEG Peak Power + Age*

S1.6 Selection of Confounders for Mediation Analysis

We created a cDAG using DAGitty.net. According to the DAGitty software, the minimally sufficient adjustment for this model only includes age as a confounder. It is important to note that many confounders were also controlled for at the level of subject selection. These included any medical illness affecting central nervous system function, any neurological disorder (other than Alzheimer's disease), history of head trauma, moderate or severe depression, and current substance abuse.

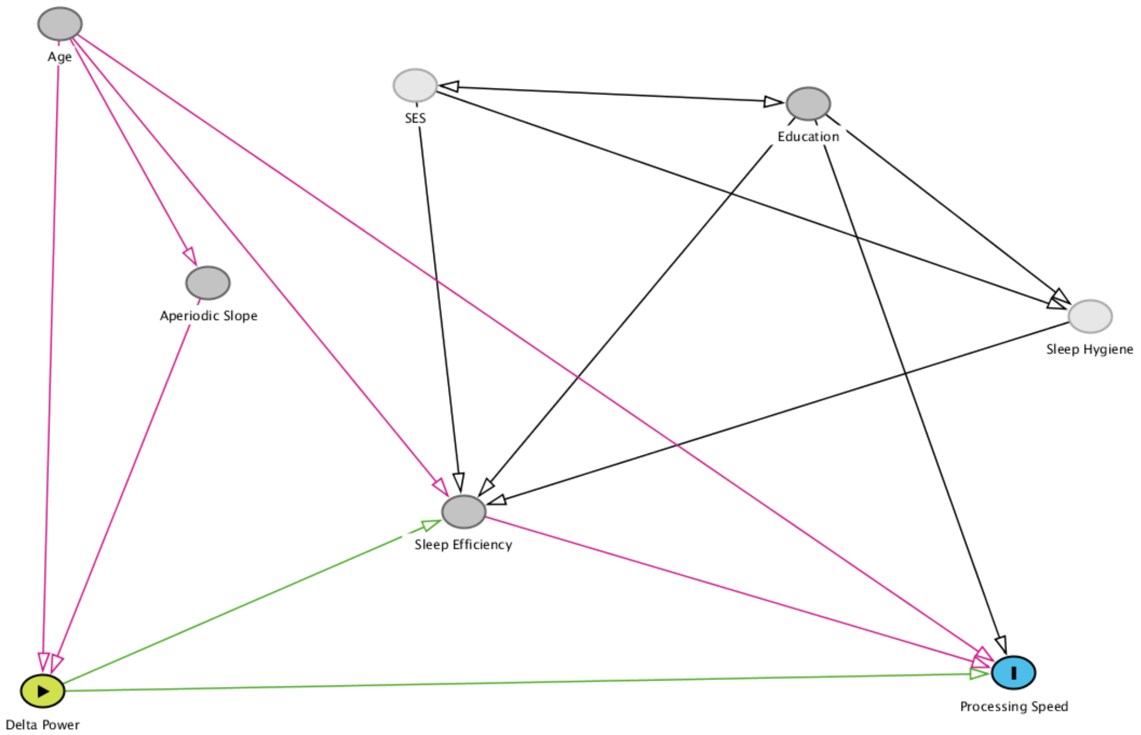

Figure S.1 cDAG Assessing Confounding Variables for Mediation Model

Supplementary Results:

S2.1 Participant Exclusions

Of the 64 participants enrolled, 62 completed the full neuroimaging and neuropsychological protocol. Four participants in the Alzheimer's Disease spectrum group were excluded after whole-brain PET imaging with florbetapir ^{18}F indicated amyloid-negativity. A total of 58 participants, 38 on the Alzheimer's Disease spectrum (aMCI, n = 17; mild probable Alzheimer's Disease, n = 21), and 20 cognitively normal controls are included in this study. One participant on the Alzheimer's Disease spectrum was found to have outlier data in the delta frequency band (power values > 4 SD from the group mean) and was thus excluded from all analyses considering delta power.

S2.2 Aperiodic-corrected Model Confirms that Sleep Efficiency Fully Mediates the Relationship between Delta Power and Processing Speed in Alzheimer's Disease

We tested whether the mediation of the delta power/processing speed relationship by sleep efficiency in patients on the Alzheimer's Disease spectrum changed upon correction for the aperiodic slope and offset. Even when controlling for variability in the aperiodic component, sleep efficiency fully mediated the relationship between delta power and processing speed, such that there was a significant indirect effect (ACME = -3.25, 95% CI [-7.87, -0.43],

p = .024), and no significant direct relationship between delta power and processing speed (ADE = 0.78, 95% CI [-6.71, 10.62], *p* = .823), once sleep efficiency was included in the model. Additionally, we performed a sensitivity analysis using medsens(), which showed that this model is robust to possible unmeasured confounders. As a secondary check, we also included all measured variables included in the cDAG (Figure S.1) into the mediation model and the pattern of results was unchanged (ACME = -3.16, 95% CI [-7.82, -0.38]).

Table S.1 *Correlations between Variables of Interest*

Variables	1	2	3	4	5	6	7
1. Age	-	-	-	-	-	-	-
2. Delta Power (peak)	-.35<br>[-.60, -.02]	-	-	-	-	-	-
3. Delta Aperiodic Slope	-.27<br>[-.54, .05]	.66<br>[.43, .81]	-	-	-	-	-
4. Alpha Power (peak)	.31<br>[-.01, .58]	-.77<br>[-.88, -.60]	-.51<br>[-.71, -.23]	-	-	-	-
5. Alpha Aperiodic Slope	-.05<br>[-.36, .28]	.46<br>[.16, .68]	.81<br>[.67, .90]	-.44<br>[-.66, -.14]	-	-	-
6. Sleep Efficiency	.05<br>[-.27, .37]	-0.44<br>[-.72, -.23]	-.39<br>[-.63, -.08]	.25<br>[-.07, .53]	-.24<br>[-.52, .09]	-	-
7. Processing Speed	.35<br>[.03, .60]	-.36<br>[-.61, -.04]	-.44<br>[-.67, -.14]	.33<br>[.01, .59]	-.28<br>[-.55, .05]	.44<br>[.14, .66]	-

Note. Estimates presented are Pearson's r-values with 95% Confidence Intervals in brackets.

Table S.2 *Standardized and Unstandardized Estimates for Mediation Paths*

Path	$\hat{\beta}$	***b***	***95% CI***	***p-value***
X-M	-.56	-52.87	[-82.75, -23.00]	.001
M-Y	.42	0.08	[0.03, 0.13]	.005
X-Y	-.29	-5.03	[-10.89, 0.83]	.090
X-Y (M included)	-.05	-0.81	[-7.20, 5.58]	.798

Note. X = Delta Power, M = Sleep Efficiency, Y = Processing Speed. All paths included age in the model as a covariate of no interest.

Table S.3 *Descriptive Statistics for MCI and AD subgroups*

Descriptives, mean (SD)	Mild Cognitive Impairment	Alzheimer's Disease	*p - value*‡
	n = 17	n = 20	
Delta Power (peak)	0.26 (0.07)	0.29 (0.09)	.641
Alpha Power (peak)	0.18 (0.05)	0.18 (0.06)	.523
Sleep Efficiency, %	96.95 (4.93)	94.85 (9.14)	.659
MMSE	26.88 (2.00)	21.95 (3.41)	<.001

‡*p*-values indicate group differences as measured by independent samples *t*-test with equal variances not assumed.

Supplementary References:

1. Tadel F, Baillet S, Mosher JC, Pantazis D, Leahy RM. Brainstorm: A User-Friendly Application for MEG/EEG Analysis. *Comput Intell Neurosci*. 2011;2011:1-13. doi:10.1155/2011/879716

2. Fischl B. FreeSurfer. *NeuroImage*. 2012;62(2):774-781. doi:10.1016/j.neuroimage.2012.01.021

3. Taulu S, Simola J. Spatiotemporal signal space separation method for rejecting nearby interference in MEG measurements. *Phys Med Biol*. 2006;51(7):1759-1768. doi:10.1088/0031-9155/51/7/008

4. Donoghue T, Haller M, Peterson EJ, et al. Parameterizing neural power spectra into periodic and aperiodic components. *Nat Neurosci*. 2020;23(12):1655-1665. doi:10.1038/s41593-020-00744-x

5. Smith S, Nichols T. Threshold-free cluster enhancement: Addressing problems of smoothing, threshold dependence and localisation in cluster inference. *NeuroImage*. 2009;44(1):83-98. doi:10.1016/j.neuroimage.2008.03.061

6. Kleiber C, Zeileis A, Zeileis MA. Package 'aer.' *R Package Version*. 2020;12(4).
